# Mechanical bowel preparation for elective colorectal surgery

**DOI:** 10.1007/s10151-019-02061-3

**Published:** 2019-08-30

**Authors:** J. Lewis, J. Kinross

**Affiliations:** Department of Surgery and Cancer, Imperial College London, London, UK

## Abridged abstract [1]

### Background

The presence of bowel contents during colorectal surgery has been related to anastomotic leakage, but the belief that mechanical bowel preparation (MBP) is an efficient agent against leakage and infectious complications is based on observational data and expert opinions only.

### Objectives

To determine the security and effectiveness of MBP on morbidity and mortality in colorectal surgery.

### Search methods

Publications describing trials of MBP before elective colorectal surgery were sought through searches of MEDLINE, EMBASE, LILACS, IBECS and The Cochrane Library. Searches were performed December 1, 2010.

### Selection criteria

Randomized controlled trials (RCTs) including participants submitted for elective colorectal surgery.

### Main results

For the comparison mechanical bowel preparation (Group A) versus no mechanical bowel preparation (Group B) results were: (1) anastomotic leakage for low anterior resection: 8.8% (38/431) of Group A, compared with 10.3% (43/415) of Group B; Peto OR 0.88 [0.55, 1.40]. (2) Anastomotic leakage for colonic surgery: 3.0% (47/1559) of Group A, compared with 3.5% (56/1588) of Group B; Peto OR 0.85 [0.58, 1.26]. (3) Overall anastomotic leakage: 4.4% (101/2275) of Group A, compared with 4.5% (103/2258) of Group B; Peto OR 0.99 [0.74, 1.31]. (4) Wound infection: 9.6% (223/2305) of Group A, compared with 8.5% (196/2290) of Group B; Peto OR 1.16 [0.95, 1.42]. Sensitivity analyses did not produce any differences in overall results. For the comparison mechanical bowel preparation (A) versus rectal enema (B) results were: (1) anastomotic leakage after rectal surgery: 7.4% (8/107) of Group A, compared with 7.9% (7/88) of Group B; Peto OR 0.93 [0.34, 2.52]. (2) Anastomotic leakage after colonic surgery: 4.0% (11/269) of Group A, compared with 2.0% (6/299) of Group B; Peto OR 2.15 [0.79, 5.84]. (3) Overall anastomotic leakage: 4.4% (27/601) of Group A, compared with 3.4% (21/609) of Group B; Peto OR 1.32 [0.74, 2.36]. (4) Wound infection: 9.9% (60/601) of Group A, compared with 8.0% (49/609) of Group B; Peto OR 1.26 [0.85, 1.88].

### Authors’ conclusions

There is no statistically significant evidence that patients benefit from mechanical bowel preparation, nor the use of rectal enemas. Further research on patients having elective rectal surgery, below the peritoneal reflection, in whom bowel continuity is restored, and studies with patients having laparoscopic surgery are still warranted.

## Commentary

Recent advances in our understanding of the biological mechanisms underlying anastomotic leak [2] have led to a re-examination of the role of bowel preparation in elective colorectal surgery. MBP is commonly with the aim of reducing peritoneal contamination and to reduce wound infections or the risk of anastomotic dehiscence. Much of the current debate has been fueled by retrospective data from US registries which suggest that oral antibiotics, when used with bowel preparation, reduced surgical morbidity [3]. However, high-quality data on the single use of mechanical bowel preparation are still lacking and as a result, there is significant clinical heterogeneity in its use. The Cochrane review on this topic from 2011 [1] is, therefore, of relevance once more. For this systematic review and meta-analysis, the authors collated both published and unpublished data from 18 heterogenous studies which, at the time of writing, provided the most rigorous data on mechanical bowel preparation. In this fourth update of the Cochrane review, including data from 5805 patients, the authors found that mechanical bowel preparation confers no advantage over either no bowel preparation or rectal enema with regard to infectious complications or anastomotic dehiscence. This lack of a statistically significant difference in outcomes holds true for both colonic and rectal resections.

However, the analysis warrants closer inspection. Of the eight studies that calculated sample size, only three were powered to detect differences in anastomotic leak [4–6]. More importantly, there is significant heterogeneity across the clinical studies both in terms of trial design and clinical intervention. For example, a variety of different bowel preparation methods were used including mannitol, bisacodyl, polyethylene glycol, and sodium phosphate. Recent data suggest that the colonic mucosal bacteria vary significantly according to the underlying pathology [7], yet these studies included heterogenous cohorts of patients having surgery for both malignant and benign conditions. Similarly, there was no standardization of surgical approach (three studies report the inclusion of laparoscopic surgery) or post-operative management; in the current era of laparoscopic surgery and enhanced recovery protocols, the results should, therefore, be interpreted with caution.

Perhaps most importantly, a variety of antimicrobial prophylaxis approaches were used which are likely to have confounded many of the central conclusions of the analysis. One study alone includes five intravenous antibiotic combinations [4], some routinely continued antibiotics (gentamicin + metronidazole) post-operatively for 24 [8] or 48 h [9] (or longer), some used for enteral (sulfamethoxazole/trimethoprim + metronidazole or doxycycline + metronidazole) antibiotic prophylaxis [5], and one used both enteral (neomycin + metronidazole 24 h pre-operatively) and intravenous (ceftriaxone + metronidazole at induction) antibiotic prophylaxis [10]. The widely varied use of antibiotics has not been controlled for in this analysis. The authors note that all studies included antibiotic prophylaxis in some form and note the importance of antibiotic prophylaxis in preventing infectious complications, but do not control for the different routes or patterns of administration in analysis.

Although no mention is made in this review of patient acceptability or complications of bowel preparation, some of the individual papers [4, 5, 11] mention the concerns of patient discomfort, bloating dehydration, nausea, and electrolyte imbalances which detract from the goal of optimal peri-operative physiology. The discomfort of mechanical bowel preparation led to 11% of patients failing to complete preparation in one study [12], and Moral et al. [10] reported that 35% of their patients described intense discomfort with mechanical bowel preparation. Platell et al. [6] noted that mechanical bowel preparation with polyethylene glycol resulted in more patient discomfort and nausea than enema preparation and that the mechanical bowel preparation was reported to be significantly worse.

The reviewers state that updates are considered every 2 years, but none has been forthcoming for 8 years. In that time, clinical practice has advanced with many centres in the UK adopting oral antibiotic use. In a more recent Cochrane review by Nelson et al. [13], which explored the use of antimicrobial prophylaxis in colorectal surgery, a significantly reduced risk of surgical site infection was identified when using combined oral and intravenous antibiotic preparation compared to intravenous alone [RR 0.55, 95% CI 0.43–0.71, *p* = 0.0001, data from 15 randomized controlled trials (RCTs) with 2929 patients] or oral alone (RR 0.52, 95% CI 0.35–0.76, *p* = 0.0003, data from 9 RCTs with 1990 patients). In fact, Bellows et al. [14] suggest a number needed to treat of 20 when using oral, non-absorbed antibiotics in addition to intravenous prophylaxis to prevent wound infections after colorectal surgery. They do add that they consider mechanical preparation of the bowel an important synergist to antimicrobial preparation to reduce the bacterial burden and improve delivery of antibiotics to the mucosa.

Perhaps the best recent evidence is an analysis of 27,804 subjects in the American College of Surgeons—National Surgical Quality Improvement Program (ACS-NSQIP) database by Klinger et al. [2]. They showed that oral antibiotic bowel preparation is protective against surgical site infection, organ space infection, and anastomotic leak when compared to no preparation. The same was not true for mechanical bowel preparation alone, which showed no significant difference in results to unprepared bowel. The combination of oral antimicrobial and mechanical bowel preparation also reduced the risk of surgical site infection when compared to oral antimicrobial preparation alone. Although this provides some evidence in support of combined mechanical and antimicrobial bowel preparation, we are still lacking a large-scale randomized trial of bowel preparation techniques to answer the question of how to proceed. While there have been several studies into the effect of antimicrobial bowel preparation, none have replicated modern practices with laparoscopic surgery and enhanced recovery after surgery (ERAS) protocols, most are not powered for leak, and they have mostly compared oral with intravenous antimicrobial prophylaxis. Moreover, few have reported mechanisms, side effect data or complications to the patient (e.g., *Clostridium difficile* infection).

The gut microbiota (the community of prokaryotes and eukaryotes colonising the gastrointestinal tract) varies between individuals, as does the microbiome (the genomic content of the microbiota) [15]. Recent work has implicated the microbiome in the pathogenesis of anastomotic leak and demonstrated that virulent strains of *Enterococcus faecalis* can take the advantage of a depleted post-operative colonic microbiome and contribute to anastomotic leak via MMP-9 activation and collagenase expression [1]. Mucosal bacteria therefore represent a legitimate target for peri-operative strategies aiming to reduce risk; however, the best method to target these bacteria is still not established and there remains a lack of precision strategies for targeting niche-specific strains. Moreover, the microbiome has a very broad range of health benefits for the host [16] and a perturbed gut ecology will influence the efficacy and toxicity of the response to adjuvant chemotherapy [17]. The importance of the microbiome is, therefore, nuanced, and it is unclear what harm may result from the attempted eradication of the entire colonic microbiome. It is believed that a diverse flora is an important constituent of health and is therefore counterintuitive to eradicate the entire flora for short-term surgical outcomes when the long-term effects of this is not yet understood.

The 2011 Cochrane review [1] remains the best evidence available on mechanical bowel preparation. However, mechanical bowel preparation is still in widespread clinical practice, despite the discomfort caused, and global targeting of the gut microbiome with antibiotics continues to gain popularity despite the lack of understanding of the role of the microbiome in anastomotic healing. More prospective data are now urgently required on the mechanisms of bowel preparation and the reasons why there are such large inter-individual variations in the response to this therapy. Moreover, we will need properly powered and designed microbiome-based trials to inform clinicians on the optimal way to reduce wound infection and anastomotic leak rates.
